# Editorial: Omics for infertility and contraception: two sides of same coin

**DOI:** 10.3389/fcell.2023.1293677

**Published:** 2023-09-26

**Authors:** Soumya Ranjan Jena, Gayatri Mohanty, Kavindra Kumar Kesari, Damayanthi Durairajanayagam, Luna Samanta

**Affiliations:** ^1^ Redox Biology and Proteomics Laboratory, Department of Zoology, Ravenshaw University, Cuttack, India; ^2^ Department of Veterinary and Animal Sciences, University of Massachusetts, Amherst, MA, United States; ^3^ Department of Applied Physics, School of Science, Aalto University, Espoo, Finland; ^4^ Research and Development Cell, Lovely Professional University, Phagwara, Punjab, India; ^5^ Department of Physiology, Faculty of Medicine, Universiti Teknologi MARA, Selangor, Malaysia

**Keywords:** male infertility, sperm chromatin, genomics, transcriptomics, proteomics, metabolomics

The Research Topic entitles “Omics for infertility and contraception: two sides of same coin” is focused on the intertwined issues of contraception and infertility. Contraception is essential for population stability, while infertility poses emotional and psychological challenges for the working-age population. There is a significant global need for family planning (57% of women), but millions lack access (United Nations et al., 2019). Conversely, about 15% of couples face infertility (Eshre, 2017). Assisted reproductive technologies (ART) offer hope but have limitations due to our limited understanding of reproductive physiology. Addressing these challenges requires prioritizing non-hormonal contraceptives (non-HPG) for both genders to avoid side effects, such as cancer risks associated with hormonal contraceptives (Hemmerling et al., 2020). It is also crucial to identify specific molecules responsible for reproductive functions. These interconnected issues benefit from advancements in each other. Additionally, OMICS technologies and bioinformatics enable comprehensive study of cellular processes from DNA to metabolites, offering strategies for non-HPG contraceptives (Johnston and Goldberg, 2020). Targeting structural proteins and interactions shows promise but requires inhibitor development. Although progress has been made in identifying biomarkers for infertility (Mohanty et al., 2020; Jena et al., 2021; Mohanty et al., 2021; Nayak et al., 2023), druggable targets remain elusive. This special volume covers recent OMICS-based advancements in understanding reproduction at molecular level not only in human but also in farm animals including the application of artificial intelligence in infertility diagnostics.

Karanwal et al. studied the significance of water buffalo (Bubalus bubalis) in India’s dairy sector and the economic losses from failed pregnancies after artificial insemination (AI), often due to low-quality bull semen. They used LC-MS/MS to analyze high fertile (HF) and low fertile (LF) buffalo sperm proteins. They found 1,385 proteins, with 1,002 shared and 288 unique to HF, and 95 to LF. HF had 211 significantly more abundant proteins, linked to vital sperm functions. In contrast, LF had 342 less abundant proteins associated with processes like glycolysis and inflammation. Fertility-related proteins (e.g., AKAP3, Sp17, and DLD) were validated, offering potential markers for buffalo fertility prediction, aiding the farming sector’s economic stability.

Ovastacin (ASTL) is a crucial protein released during fertilization, preventing polyspermy and aiding embryo protection. Deleterious SNPs in ASTL can lead to female infertility by disrupting its interactions with ZP2 and FETUB (Xiong et al., 2017). In this study, 4,748 SNPs were analyzed by Suri et al., with 40 ns SNPs identified in ASTL’s catalytic domain. MutPred2 indicated changes in catalytic activity/zinc binding. Docking studies revealed important hydrophobic and hydrogen bonding interactions between ASTL, ZP2, and FETUB. Notably, a cluster of SNPs occurred in the conserved motif 198DRD200. Statistical Coupling Analysis (SCA) confirmed the significance of these SNPs in functionally critical positions of ASTL. These findings highlight regions in ASTL susceptible to mutations that could cause female infertility.

This study reported by Oluwayiose et al. demonstrated the role of non-coding RNAs (ncRNAs) in the seminal plasma extracellular vesicle (spEV) in male infertility cases with and without successful live births after assisted reproductive technology (ART) treatment. They analyzed small RNA profiles from 91 semen samples, categorizing couples into live birth (28) and no live birth (63) groups. They found 12 differentially expressed spEV ncRNAs, including 10 circRNAs and two piRNAs. Most circRNAs were downregulated in the no live birth group, linked to reproductive and developmental processes. The upregulated piRNAs overlapped with genes related to mitochondrion morphogenesis, signal transduction, and cellular proliferation. These findings highlight the male partner’s role in ART success.

The opinion article by Sengupta et al. highlights the advancement in application of artificial intelligence and deep learning in basic semen analysis, the gold standard prescribed by World Health Organization (WHO) for male infertility evaluation. Mojo-AISA (Automated *In situ* Semen Aanlysis), is the first microscope integrated with an artificial intelligence algorithm. This article analyses the potential of Mojo-AISA to enhance accuracy and efficiency in diagnosing male infertility. Mojo AISA uses neural networks to determine sperm concentration and motility accurately. It was evaluated against manual microscopy methods using 64 semen samples and showed the ability to provide precise results in half the time. However, challenges include assessing samples with very low concentration and the need for correct slide preparation. Despite limitations, Mojo AISA may improve semen analysis accuracy, reduce inter-laboratory variability, and boost productivity in embryology labs, benefiting reproductive medicine.

These approaches enable personalized medicine, biomarker discovery, and safer, more effective contraception methods while linking infertility and contraception advancements.
